# Obstacles on the way to the clinical visualisation of beta cells: looking for the Aeneas of molecular imaging to navigate between Scylla and Charybdis

**DOI:** 10.1007/s00125-012-2491-7

**Published:** 2012-02-23

**Authors:** K. Andralojc, M. Srinivas, M. Brom, L. Joosten, I. J. M. de Vries, D. L. Eizirik, O. C. Boerman, P. Meda, M. Gotthardt

**Affiliations:** 1 Department of Nuclear Medicine, Radboud University Nijmegen Medical Centre, PO Box 9101, 6500 HB Nijmegen, the Netherlands; 2 Department of Tumour Immunology, Radboud University Nijmegen Medical Centre, Nijmegen, the Netherlands; 3 Laboratory of Experimental Medicine, Université Libre de Bruxelles, Brussels, Belgium; 4 Deparment of Cell Physiology and Metabolism, University of Geneva, Geneva, Switzerland

**Keywords:** Beta cell mass, Insulin, Insulitis, Islets of Langerhans, MRI, Pancreas, PET, Radiochemicals, Review, SPECT

## Abstract

For more than a decade, researchers have been trying to develop non-invasive imaging techniques for the in vivo measurement of viable pancreatic beta cells. However, in spite of intense research efforts, only one tracer for positron emission tomography (PET) imaging is currently under clinical evaluation. To many diabetologists it may remain unclear why the imaging world struggles to develop an effective method for non-invasive beta cell imaging (BCI), which could be useful for both research and clinical purposes. Here, we provide a concise overview of the obstacles and challenges encountered on the way to such BCI, in both native and transplanted islets. We discuss the major difficulties posed by the anatomical and cell biological features of pancreatic islets, as well as the chemical and physical limits of the main imaging modalities, with special focus on PET, SPECT and MRI. We conclude by indicating new avenues for future research in the field, based on several remarkable recent results.

## Introduction

**Fig. 1:**
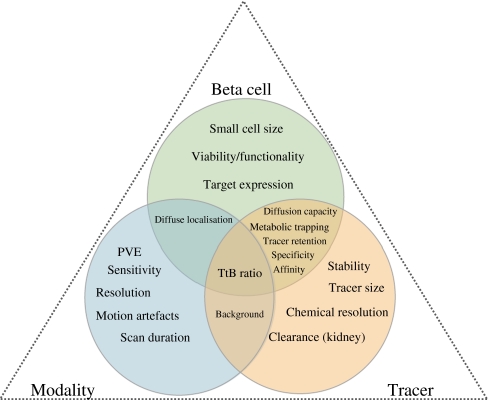
The three main groups of obstacles encountered on the way to clinical beta cell imaging: modality, tracer and beta cells themselves. PVE, partial volume effect; TtB, target-to-background ratio

Our current knowledge about the beta cell mass (BCM) in normal individuals and diabetic patients largely relies on autopsy data [1]. By necessity, these are single time-point evaluations. It is important to develop a non-invasive means of monitoring BCM as a function of time, to better understand the development and course of type 1 and type 2 diabetes, and to evaluate the effects of novel candidate glucose-lowering drugs, which may modify the BCM [2]. Researchers have been tackling this problem for about 15 years. Since then, the National Institutes of Health (Bethesda, MD, USA) have organised four workshops on beta cell imaging (BCI) [3]. The first European workshop on BCI took place in Stockholm, Sweden, on the occasion of the annual EASD meeting in 2010. Despite this drive, and some promising initial observations [4], progress has been hindered by many problems, so that only one tracer (dihydrotetrabenazine [DTBZ]), which targets vesicular monoamine transporter 2 (VMAT2), is currently under clinical evaluation for positron emission tomography (PET) imaging of pancreatic islets [5]. Of concern, quantitative measurement of the signal for this tracer remains challenging [6], and questions about the suitability of the target and the specificity of the tracer remain [7]. To many diabetologists it may not be clear why the imaging world struggles to develop a simple and effective method for clinical, non-invasive BCI, especially since developments towards the molecular characterisation of tumours and techniques for imaging the consequences of metabolic disorders have become a reality in other biomedical fields. Here, we review the obstacles hindering the development of clinical BCI (Fig. 1).

A first challenge in the quest to adapt existing techniques for BCI is that the target is a diffuse collection of cell clusters, dispersed throughout the pancreas, that constitutes less than 2% of the total mass of the adult gland. This volume is likely to decrease during the course of diabetes [2]. Therefore, BCI requires either a high spatial resolution or a high ‘chemical resolution’, meaning, a highly specific tracer molecule that targets beta cells but not the surrounding exocrine pancreas. Present anatomical clinical imaging modalities, such as computed tomography (CT) or clinical MRI, cannot resolve individual islets of Langerhans, which typically range from 20 to 600 μm in diameter. On the other hand, functional clinical imaging modalities with very high sensitivity, such as PET or single photon emission computed tomography (SPECT) are hampered by the partial volume effect, leading to an underestimation of the signals derived from objects smaller than the spatial resolution of the scanner. Another problem is that imaging modalities have either a high sensitivity (SPECT, PET) or a high spatial resolution (CT, MRI), but rarely have a combination of both characteristics in a clinically useful mode. Furthermore, the sensitivity of tracer-based imaging is dependent on the level of expression of the target. In radiotracer imaging of tumours, the target is usually overexpressed on the tumour cells in comparison to the healthy tissue, thus leading to higher accumulation [8–10]. This is also the case for the endocrine pancreas. In insulinomas, for example, the density of the glucagon-like peptide 1 receptor (GLP-1R) is considerably higher than in normal pancreas (mean density of 8,302 ± 1,073 dpm/mg in benign human insulinomas vs. 1,563 ± 104 dpm/mg in normal endocrine pancreas) [10, 11]. This difference presumably explains why tracers such as [^18^F]fluoro-l-dihydroxyphenylalanine ([^18^F]-DOPA) that are suitable for imaging insulinomas and nesidioblastosis by targeting D_2_ receptors [12] are not adequate for imaging the native beta cells.

Other issues concern the specificity, affinity (affinities sufficient for therapy may not suffice for imaging) and size of tracer molecules (large molecules are retained in blood and have a lower diffusion capacity, resulting in low target-to-background ratios). Several more hurdles, such as the site of transplantation, confront the imaging of transplanted islets. For metabolic and physiological reasons, the liver is the preferred site for islet transplantation, but their diffuse distribution throughout the sinusoid vascular bed vastly complicates the detection of the grafted islets. When islets are pre-labelled, as is generally the case for MRI [4], large loads of label are required, which may alter islet viability and function.

Thus, it is evident that in vivo BCI is challenging, and that each imaging technology is associated with a number of trade-offs, owing to the nature of the imaging equipment, the tracer molecules used and the characteristics of the islets of Langerhans (see text box). The situation recalls that faced by the mythical Greek heroes Odysseus, Jason and Aeneas, navigating between Scylla and Charybdis. Each of them chose different solutions: Odysseus sacrificed some of his sailors, Jason asked for the help of goddess Hera and Aeneas pushed through with brute force and luck. It is still uncertain which approach, or combination thereof, will work for BCI. In the following sections, we discuss these limitations and the challenges faced by the different imaging modalities.
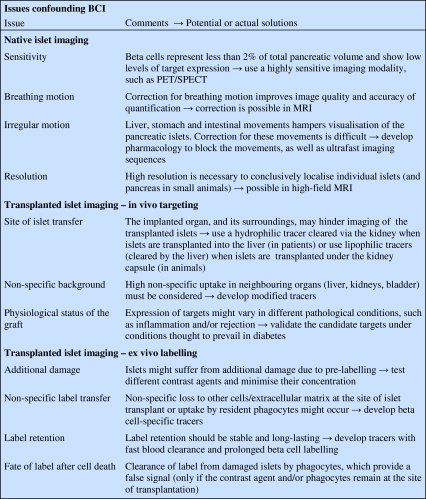


## Radionuclide imaging (PET/SPECT)

Radionuclide imaging is based on the specific uptake of a radiolabelled tracer, and the subsequent detection of γ photons emitted by the decaying nuclide. Nuclear medicine imaging (NMI) techniques such as PET and SPECT provide three-dimensional images of the distribution of the tracer. Moreover, NMI accurately quantifies radioactivity concentrations in the picomolar range and provides information on the kinetics of tracer distribution. In contrast, the performance of PET and SPECT is comparatively poor in terms of spatial resolution (>1 mm). Thus, it is still not possible to visualise single pancreatic islets with NMI, especially in clinical scanners, where PET performs better (spatial resolution of 2–4 mm) than SPECT (spatial resolution of 8–10 mm) and the determination of BCM requires high accumulation of a radiotracer that specifically labels beta cells.

### Native islets

For in situ detection of native islets, it has been estimated that the uptake/binding of a tracer should be 1,000 times higher in beta cells than in other cell types [13]. For example, the dopamine D_2_-like receptor was considered an attractive target [14], but attempts to determine the BCM by [^18^F]-DOPA PET failed because of the high uptake of the tracer in the exocrine pancreas, limiting the usefulness of this tracer to detect focal nesidioblastosis [15, 16]. In the latter situation, high numbers of insulin-producing cells form clusters that are larger than most islets, and these occupy a substantial fraction of the neonatal pancreas. As an alternative, targeting VMAT2 with radiolabelled DTBZ has now been tested. In humans, VMAT2 is present in beta cells [17, 18] and in the sympathetic nerves throughout the gastrointestinal tract and pancreas [19]. Thus, despite specific VMAT2 targeting in vitro, a high and non-specific uptake of radiolabelled DTBZ has been observed in the exocrine pancreas [7, 19–21]. This important non-beta cell binding may explain the modest decrease in DTBZ uptake observed in long-term type 1 diabetic patients and in rodent models of the disease. In this case a 50% decrease in binding is observed relative to healthy controls, in spite of the fact that at least 90% of the beta cells are lost [6, 22–24]. Therefore, it is unlikely that the tracer could detect the small differences in BCM anticipated in type 2 diabetes. Ideally, uptake by other endocrine cells of the islets should also be minimal. The sulfonylurea SUR1 receptor, which has been considered for determination of BCM [25, 26], is expressed by all islet cell types, and thus may give ambiguous results. Furthermore, several radiolabelled SUR1 ligands have low and non-specific uptake in the pancreas and high uptake in adjacent organs [27, 28].

GLP-1R is mostly expressed on beta cells, with lower levels of expression on alpha and gastric parietal cells [29]. Exendin, which is a natural ligand of GLP-1R, is extensively taken up by islets and less by the exocrine pancreas. Unfortunately, kidney uptake of exendin is also high [30]. Preliminary data on the determination of the BCM in rat models of diabetes using GLP-1R targeting are promising [31]. Also, Reiner et al [32] showed that GLP-1R could be targeted by exendin-4 conjugated with a near-infrared fluorophore for intravital fluorescent imaging [32], which supports the idea of GLP-1R imaging as a valid approach.

As mentioned above, radiotracers with high specific activity should result in a high beta cell-to-background ratio. However, even if a radiotracer is highly specific, this does not automatically translate into target identification in scintigraphic scans. For instance, when clearance from the blood is low, non-specific signal from circulating tracer may hamper the detection and quantification of the specific signal from beta cells. Therefore, both a high radioactive uptake ratio of endocrine-to-exocrine pancreas and a high radioactive uptake ratio of pancreas-to-surrounding organs and blood are necessary. For example, the monoclonal islet cell surface antibody IC2 specifically targets beta cells, and the uptake of ^125^I-labelled IC2 correlates linearly with BCM in the mouse [33]. However, the slow clearance of this monoclonal antibody from the blood, which is due to the large immunoglobulin size, is likely to limit its application in humans. F(ab′)_2_ fragments, single chain antibodies, nanobodies and Affibodies may be interesting alternatives, since they have specificities and affinities towards the target that are similar to those of native antibodies but have a faster clearance as the result of a much smaller size. Unfortunately, most of these immunoglobulin forms feature high kidney uptake. Another alternative is the single-chain antibodies produced by phage-display technology, which show high uptake in the rat pancreas, a linear correlation with BCM and fast blood clearance [34].

Excretion routes also significantly affect the target-to-background ratios. Hydrophilic molecules are generally cleared via the kidneys, whereas lipophilic and large molecules are cleared via the hepatobiliary system. Renal clearance can result in unspecific tubular absorption of radiolabelled peptides, and the close proximity to the pancreas may hamper BCI [35]. Co-injection of lysine, poly-glutamic acid or a gelatine-based plasma expander together with the radiolabelled tracer reduces, but does not completely block, unspecific renal uptake [35]. Clearance via the hepatobiliary system results in high accumulation of tracers in the liver and might also result in excretion via the intestinal organs, leading to enhanced radioactivity concentrations in the intestinal tract. In turn, high intestinal uptake will decrease the pancreas-to-background ratio. This is the case for ^11^C- and ^18^F-labelled DTBZ, which are under clinical evaluation [7, 19–21]. The introduction of a hydrolysable epoxide shifts the route of excretion from the liver to the kidney, resulting in lower accumulation in non-target organs [36].

Another important aspect of an ideal tracer for BCI is the specific activity, i.e. the amount of radioactivity (usually expressed in becquerels, Bq) attached to a given amount of tracer molecule (usually expressed in moles). The high activity doses that are required for imaging the small sized islets, may temporarily block the target and reduce its tracer accumulation [37, 38]. In the case of exendin, the optimal dose for targeting GLP-1R is 20 pmol [38]. This requires a specific activity of more than 500 GBq/μmol to enable BCI by SPECT, using ^111^In-DTPA-exendin [39]. This is in contrast to pharmacological approaches, where an excess of certain drugs is often applied to achieve the maximum therapeutic effect. Moreover, tracers that require higher injected doses may cause side effects, especially when biologically active compounds/derivatives (e.g. GLP-1 analogues or SUR1 agonists) are used.

Tracer stability also plays a major role in successful imaging, since chemically unstable molecules have low or no accumulation in target cells. For instance, ^125^I-labelled dithizone, a chelator of heavy metals, which stains beta cells in vivo owing to their high zinc content [40], is rapidly cleaved, releasing ^125^I in biological fluids [41]. Conjugation of histamine increases the stability of the radio-iodinated dithizone, but this modification results in high uptake in the liver and exocrine pancreas [42, 43], making this approach useless for BCI. Most peptides are prone to degradation by endogenous peptidases, usually resulting in lower receptor affinity and/or biological activity. For example, somatostatin has a plasma half-life of less than 3 min in humans [44] and the introduction of d-amino acids and a reduced C-terminus increases the plasma half-life of the somatostatin analogue octreotide [44–47]. These modifications have made octreotide a suitable ligand for the somatostatin receptor, leading to its widespread use in the imaging of neuroendocrine tumours [48, 49]. However, structural modifications of peptides often reduce their binding affinity and/or internalisation kinetics and, thus, tracer uptake. Prevention of peptide degradation can also be achieved by conjugation to a large protein, such as albumin. As an example, GLP-1 conjugated to albumin prolongs GLP-1R signalling [50]. However, this conjugation results in slower clearance as a result of the larger size of the complex, leading to a lower pancreas-to-background ratio.

### Transplanted islets

Transplantation of pancreatic islets of Langerhans is a promising treatment for type 1 diabetic patients. The ability of islet transplantation to normalise blood glucose levels is encouraging [51], even though the rate of insulin-independency drops to less than 15% after 5 years. In view of the considerable side effects caused by the immunosuppressive therapy required in islet transplantation, a more favourable outcome should be reached. A method that would allow us to specifically visualise transplanted beta cells in vivo in humans could be a key factor in improving the outcome of islet transplantation. If such a method could monitor the islet graft function, which to date can only be indirectly estimated by blood glucose, insulin and C-peptide levels, a relationship between BCM and beta cell function could be determined.

Results of the first successful in vivo imaging of an islet graft were published in 2004 [52]. Islets were transfected with an adenoviral vector coding for the luciferase gene [52]. A similar approach was used by the same group, using PET. In this case, the islets were transfected with a reporter gene, leading to trapping of the PET probe in islet grafts, which could then be visualised in vivo [53]. In another study, PET was also successfully used for imaging islets that expressed a reporter gene under the control of the insulin promoter. The signal obtained by PET directly correlated with insulin production [54]. Although this groundbreaking work has shown the feasibility of in vivo imaging of transplanted beta cells, the necessity to either genetically modify the beta cells or to infect them with viruses currently limits the use of these technologies to animal experimentation. In another experiment, post-transplantation events could be monitored for up to 6 h following ex vivo pre-labelling of islets with [^18^F]fluorodeoxyglucose ([^18^F]-FDG) [55]. However, the method does not allow for long-term follow-up, given the short half-life of ^18^F (110 min) and the possibility of pre-labelling islets only once. In the optimal situation the tracer should target beta cells in vivo, enabling multiple imaging sessions in the same individual at multiple defined time-points.

Despite the overall progress of these approaches, imaging of transplanted islets remains technically demanding in humans. Indeed, the existing techniques require manipulation of the islets prior to transplantation for pre-labelling, genetic modification or viral infection. Therefore, there is an urgent need to develop a tracer that would enable simple, reproducible and safe in vivo imaging of transplanted beta cells in patients.

## MRI

MRI has several advantages over NMI, including relatively easy clinical application, no involvement of radioactive isotopes and suitability for both repeated and long-term imaging. MRI has high spatial resolution, permitting up to single cell imaging under appropriate conditions [56, 57]. Furthermore, MRI is not limited by tissue penetration and shows endogenous soft tissue contrast, thus providing exquisite anatomical context. However, MRI is less sensitive than NMI techniques and is therefore presently more adaptable to imaging pre-labelled islets rather than native islets in situ. This is because pre-labelling typically achieves higher label concentration in the relevant cells, with lower background because of the lack of non-specific uptake. MRI sensitivity depends on several factors, including the imaging hardware, the sample homogeneity, the amount of contrast agent loaded in the cells, the imaging sequences used, the density of cells and, in some cases, the intracellular localisation of the contrast agent. The useful concentration of a contrast agent may therefore vary, although nanomolar concentrations are usually required for superparamagnetic iron oxide particles (SPIOs). MRI acquisition can be slow when high resolution is required and is therefore susceptible to motion artefacts. While the effects of breathing motion can be prevented by synchronising the image acquisition with the respiratory cycle, abdominal and peristaltic motion are more difficult to correct [58].

### Native islets

Direct imaging of unlabelled islets is not yet possible using standard clinical systems owing to limited resolution (beyond the average islet size) and the poor differential contrast of islets compared with the surrounding pancreatic tissue. Therefore, a first strategy is to use indirect means to evaluate BCM and islet function. For example, blood oxygen level dependent (BOLD) and arterial spin labelling (ASL) imaging do not require the use of external contrast agents and can be quantified [59, 60]. So far, however, the early success in preclinical models has not been reproduced in clinical studies [61]. Therefore, other approaches are now being developed to label islets in situ, prior to MRI.

One such approach uses dynamic MRI techniques to measure blood flow and vessel permeability. Changes in vasculature are expected to relate to changes in islet function and revascularisation after transplantation. The use of MRI to study vascularisation was originally developed to study tumour vascularisation in vivo. Microvascular leakage measurement using injectable SPIOs can detect pancreatic inflammation and its reversal after immunosuppressive therapy [62]. A recent study in mice using dynamic gadolinium-enhanced contrast imaging found that peak signal enhancement (a measure of vascularisation) occurred 7 days after islet transplantation in the liver. In this case the MRI data correlated with the neovascularisation of the grafts, as assessed by histological analysis [63]. Increased manganese levels result in a change in contrast [57, 64], which enables visualisation of individual islets under experimental 14.1 T field conditions [57]. Whether the manganese contrast informs about beta cell function, remains to be fully validated. However, the injection of contrast agents at larger doses than those used for ex vivo cell labelling may be toxic, possibly compromising clinical use. Recently, a small scale human trial exploited the changes in vascular permeability brought about by insulitis in individuals with recent-onset diabetes [65]. Intravenously infused SPIOs preferentially accumulated in the pancreas of patients with insulitis as a result of increased vascular leakage and macrophage uptake. The study also revealed differences in pancreatic volume between type 1 diabetic patients and healthy controls. These findings are in line with the results of previous studies that used [^18^F]-FDG PET to detect insulitis in a mouse model of type 1 diabetes [66] and in humans [67].

### Transplanted islets

Isolated islets can be pre-labelled with large amounts of contrast agents, prior to transplantation. However, labelling can be difficult since islet cells have modest phagocytic activity. Furthermore, the nature (e.g. metallic particles) and large size of some MRI labels (e.g. liposomes >300 nm in diameter) can further hinder uptake and affect islet function and viability. Label uptake can be enhanced using contrast agent with special coatings [68, 69], specific antibodies [70], encapsulation of the agent in specially formulated liposomes [71] or binding to positively-charged peptides loaded by electroporation [72]. Several of these approaches are not easily translatable to the clinic owing to their experimental nature, or financial or logistical difficulties. Direct labelling is possible using gadolinium or iron oxide chelates [73, 74]. Recent findings, however, indicate that heavy metal chelates can be toxic in humans, and this may restrict their clinical use [75]. A further approach used to greatly increase label uptake is to encapsulate the islets in permeable capsules together with the MRI agent [76–79]. However, the actual status of the islets within the capsules and their long-term fate is not known.

MRI contrast agents, most often iron oxide or gadolinium chelates, function by affecting the local contrast of an image. Typically, iron oxide results in regions of hypointensity (dark spots), while gadolinium results in regions of hyperintensity (bright spots). In general, SPIOs show higher sensitivity than other contrast agents. Accordingly, SPIO particles have successfully been used for clinical cell tracking in melanoma patients [80]. Since MRI contrast agents modify local contrast, they are greatly affected by the in vivo background. This is a major hurdle in their clinical use, especially in sites such as the liver, which are intrinsically rich in iron and already hypointense (i.e. dark in typical MRI images). Indeed, in one study using SPIO-labelled islets transplanted to the liver in humans some patients had to be excluded due to their endogenously hypointense liver, although it was possible to detect iron-induced dark spots up to 6 months after islet transplantation in other patients [81]. Moreover, local image contrast can change in response to various factors, including inflammation, bleeding or oedema, further complicating positive localisation of transplanted islets.

Another problem is the quantitative analysis of MRI images obtained with contrast agents. Contrast agents are not inherently quantitative, and spots on images may reflect islet aggregates rather than single islets. This was documented by a study comparing in vivo MRI data with histology and electron microscopy [82], which found that absolute quantification of BCM was not possible with SPIOs [83]. Even if islets could be successfully imaged in the liver and kidney capsules for a period of 30 days, some of the dark spots observed at later time points were due to extracellular iron aggregates and islets that were labelled but damaged [82]. A further and relevant issue in terms of quantification is saturation, which can occur even at relatively low concentrations of contrast agents [84]. In conclusion, the interpretation of data on labelled islets transplanted in the liver is complex, due to possible false-positives and contrast saturation issues. Special imaging sequences can be used to generate hyperintense regions after use of SPIOs [84], which typically induce hypointense spots. The hyperintense appearance improves the visualisation and definition of structures, even though contrast change remains subject to the saturation effect mentioned earlier. Therefore the technique is most applicable to low numbers of islets.

An alternative to contrast agents is ^19^F MRI. This technique is not influenced by tissue background and allows absolute quantification from in vivo images without signal saturation at higher concentrations [85, 86]. For example, diabetogenic, ^19^F-labelled T cells have been tracked and quantified as they homed to the pancreas [87]. The approach is still in the early stages of development and has not yet been applied to clinical imaging, although clinically applicable agents are being tested [88]. The ^19^F signal is easily identified and quantified, but the concentration of label required for detection is about as high as that for paramagnetic contrast agents, such as gadolinium chelates, and is several orders of magnitude higher than for supermagnetic agents, such as iron oxides [88]. Total imaging time is also longer with ^19^F MRI.

It is important to consider that labels may be taken up by different islet cells, e.g. only beta cells vs several endocrine and non-endocrine cell types, or even retained within the islet capsule. As MRI resolution is seldom sufficient to visualise single islets, the pattern of intra-islet labelling may not significantly affect the data interpretation, provided that it quantitatively reflects BCM and/or beta cell function. Evaluation of these variables requires parallel microscopy on either excised tissue sections or isolated islets labelled prior to transplantation, as well as secretion studies on the latter islets. Whenever the label may not be directly visible by microscopy, a bimodal agent such as a fluorescent nanoparticle [89] is advantageous.

Damage to liver tissue after islet transplantation is detectable by imaging and may provide indirect information about the viability of the islets. For example, intraportal islet transplantation triggers hepatic steatosis, probably caused by the locally high insulin secretion [90, 91]. Although the link between steatosis and graft function remains unclear, it appears that patients who develop fatty livers require more intense exogenous insulin therapy. Liver steatosis can also be observed using ultrasound, which is a much cheaper alternative to MRI. Furthermore, damage to the hepatic portal vein after islet transplant can also result in detectable liver ischaemia [58] and embolism of the portal vein in humans after islet transplantation [92]. Lastly, the recent introduction of responsive MR agents (chemical exchange saturation transfer [CEST] or paramagnetic chemical exchange saturation transfer [PARACEST]) also holds great promise for islet imaging. These agents can be used to detect tissue variations in pH, redox state, oxygenation or metabolite levels [93–95].

## Future directions

The data and considerations summarised above show that much remarkable progress has been made towards the in vivo imaging of native and transplanted islets during the last 15 years of research on this topic. Several approaches are already available for basic research studies of animal models, which allow direct visualisation of individual islets, including those that do not require any pre-labelling [96], or provide sound quantitative estimates of BCM in an almost fully non-invasive way [97]. These methods can help to advance our understanding of unresolved questions on the natural history of type 1 and type 2 diabetes. Yet, none of these approaches is simple and combines all the methodological and practical features that are required for a ready-to-go approach to BCI. Furthermore, even if the existing methods have provided the necessary proof of principle of concepts and methodological steps, their translation to the clinic is hindered by several ethical, safety and technological factors and is complicated by the specific histological and anatomical features of the human pancreas. To outline where the field should be going, we thought to review here the major obstacles as well as conceivable future directions for this undertaking.

There is an urgent need to develop a beta cell-specific tracer that features an acceptable bioavailability to pancreatic islets once injected into the circulation. As stressed above, such an ideal tracer is not yet available, and many independent efforts are under way to develop and validate potential tracers. Ideally, such a tracer should target a molecule that is abundant at the surface of beta cells or, alternatively, be incorporated by them. To be useful in clinical imaging, the target molecule should not be affected by the chronic inflammatory environment that is typical in type 1 diabetes. The search for candidates has, so far, been based on the screening of genomic, single nucleotide polymorphism and proteomic beta cell databanks, and this has not yet identified an ideal candidate [98]. An exciting avenue of research is the screening of all transcribed mRNAs (including splice variants of each gene) to generate splice junction databases, an approach that has already identified some beta cell-specific variants [99]. Of note, inflammatory mediators may also modify the splicing of beta cell genes [100], which opens interesting possibilities for the imaging of inflamed beta cells. Another alternative is the simultaneous targeting of multiple molecules on the beta cell surface using many specific ligands, on the assumption that the stoichiometry and distribution of the targets on beta cells are not shared by other cell types. A further interesting development is the multimerisation of the selected probes, which is expected to enhance the affinity of the ligand–target interaction, as well as the internalisation of the target–ligand complex [101]. In this approach, multiple copies of different ligands would be taken via the circulation to the beta cells by a biodegradable scaffold that could be labelled for detection by multiple imaging modalities [102]. A combination of the three latter innovations could allow the construction of the, so far, elusive beta cell-specific probe.

Another challenging step is the production of suitable ligands. Ideally, one would like to retain the exquisite affinity and specificity of antibodies, although these tend to have limited pancreatic islet access because of their large size and limited vascular extravasation. From this perspective, interesting experiments are under way to produce smaller Ig or Ig-like molecules, such as single chain antibodies, Affibodies or camelid nanobodies [103]. Given that safety and cost issues may hinder the use of antibodies or Ig-like molecules in clinical studies, a painstaking, but promising alternative is the search for other types of small molecule that exhibit better tolerance and accessibility and cost less. With the notable exception of DTBZ [5], whose beta cell specificity is already much discussed [7], the results of such a search has so far been limited. The use of high-throughput methods to screen for large numbers of compounds in cultures of insulin-producing or unrelated cells is expected to change this situation. The search for such ligands, whatever their chemical nature, should be fuelled by the large number of physiological and cell biological studies that investigate insulin secretion, as well as those concerning beta cell survival, proliferation and apoptosis. Indeed, the ultimate aim will be to image BCM and beta cell function, possibly in a single imaging session. Thus, candidates should be tested for their short- and long-term effects on different aspects of beta cell function. To date, only manganese has entered the first steps of such a screening [57, 104, 105]. However, the respective effects of BCM and beta cell function on the manganese-induced MRI signal remain to be established. The comparison of animal models featuring obvious differences in beta cell function in the presence of a similar BCM will be instrumental to unambiguously address this question.

With regard to imaging approaches, it is evident that the different characteristics of clinically adaptable techniques impose their combination in a multimodality imaging strategy. Only such a combined approach is likely to overcome the shortcomings of the individual methods, in order to provide coherent and quantitative information on both BCM and function. This will require the adaptation of the existing equipment, raising substantial technological and image analysis challenges. The recent introduction of responsive MR agents (CEST, or PARACEST) holds great promise [93–95].

As in all innovative scientific endeavours, it is not possible to make safe predictions of the outcome of these developments and the time-line they will require to be implemented. The close collaboration between islet biologists, diabetologists, radiologists, nuclear medicine physicians, radiochemists and physicists, which is essential for progress towards in vivo BCI, will undoubtedly generate much novel information about beta cell biology and diabetes. It is therefore most appropriate that such an integrative effort is now being facilitated worldwide, particularly in Europe, by several devoted programmes and sponsored by the main scientific, national and international foundations.

## Abbreviations

BCI: Beta cell imaging
BCM: Beta cell mass
CEST: Chemical exchange saturation transfer
CT: Computed tomography
[^18^F]-DOPA: [^18^F]Fluoro-l-dihydroxyphenylalanine
DTBZ: Dihydrotetrabenazine
[^18^F]-FDG: [^18^F]Fluorodeoxyglucose
GLP-1R: Glucagon-like peptide 1 receptor
NMI: Nuclear medicine imaging
PARACEST: Paramagnetic chemical exchange saturation transfer
PET: Positron emission tomography
SPECT: Single photon emission computed tomography
SPIO: Superparamagnetic iron oxide particle
VMAT2: Vesicular monoamine transporter 2
